# Daytime Sleepiness among Medical Colleges' Students in Jordan: Impact on Academic Performance

**DOI:** 10.1155/2022/7925926

**Published:** 2022-03-10

**Authors:** Mohammad Alqudah, Samar A. M. Balousha, Abedallah A. K. Balusha, Doa'a Ghazi Al-U'datt, Rami Saadeh, Nasr Alrabadi, Karem Alzoubi

**Affiliations:** ^1^Department of Physiology and Biochemistry, College of Medicine, Jordan University of Science and Technology, Irbid, Jordan; ^2^Department of Physiology, College of Medicine and Medical Sciences, Arabian Gulf University, Manama, Bahrain; ^3^King Abdullah II University Hospital, College of Medicine, Jordan University of Science and Technology, Irbid, Jordan; ^4^Department of Public Health, College of Medicine, Jordan University of Science and Technology, Irbid, Jordan; ^5^Department of Pharmacology, College of Medicine, Jordan University of Science and Technology, Irbid, Jordan; ^6^Department of Pharmacy Practice and Pharmacotherapeutics, College of Pharmacy, University of Sharjah, Sharjah, UAE; ^7^Department of Clinical Pharmacy, College of Pharmacy, Jordan University of Science and Technology, Irbid, Jordan

## Abstract

**Introduction:**

Sleep disorders are extremely prevalent in the general population. College students are more susceptible to sleep problems. This is due to the increased competition in getting a job position and the current alterations in the labor market. Poor sleep is prevalent and has deleterious effects on college students, but its frequency among college students has not been documented in Jordan. So, the aims of this study are to assess the prevalence of daytime sleepiness among medical college students in Jordan and to look for any links between daytime sleepiness and academic performance.

**Methods:**

A cross-sectional study performed on medical and paramedical specialties students and Epworth sleepiness Sscale (ESS) was used. To assess the students' academic performance, the cumulative grade point average was utilized.

**Results:**

977 students from five medical colleges participated in the study. ESS scores were abnormal in 34.4% of students and were considered to have daytime sleepiness. Significant lower ESS scores were associated with students who reported good sleep quality than students who reported poor sleep quality. Significant lower ESS scores were reported by students who slept more than 7 hours compared with students who slept less than 6 hours. The ESS scores were not significantly associated with students' CPGA.

**Conclusion:**

Daytime sleepiness is highly prevalent among medical students in Jordan. The data of this study might be very helpful to assess the academic policy makers to develop intervention strategies that resolve the sleep disturbances in college students and reduce its impact on the academic achievements.

## 1. Introduction

Sleep is a complicated physiological process that is also one of the most important human beings' behaviors. It occupies about one-third of human life [1]. Sleep is a process of the brain, and it is needed for its adequate functioning. It is thought that sleep is important for energy conservation, thermoregulation, and metabolic satisfaction [2]. Sleep consists of two phases that alternate with one another throughout the sleep cycle. These include nonrapid eye movement (NREM) and rapid eye movement (REM). The NREM made up of 3 stages, N1, N2, and N3. Sleep is initiated at the NREM sleep. REM phase is distinguished by the clear and colorful dreaming and decreased muscle tone. Sleep is easily disrupted during stage N1 of the NREM sleep. Also, the duration of this phase is increased in people who suffer from sleep deprivation {[3] #134; [1] #133; [4] #157}.

Sleep pattern differs in different subjects. It varies with age, occupation, physiological and psychological states, and some types of psychiatric and physical illnesses [5, 6]. Most normal adults sleep on average 7.5 to 8.5 hrs per sleeping cycle [7].

Sleep deprivation adversely impact life quality and is linked with an increased occurrence of systemic diseases [8, 9]. Also, prolonged periods of lack of sleep might cause delusions and hallucinations [2, 3].

Many systems aimed to classify sleep-wake disorders. Of the most famous are the Diagnostic and Statistical Manual of Mental Disorders (DSM-5), International Classifications of Sleep Disorders (ICSD-2), and International Statistical Classification of Diseases and Related Health Problems (ICD-10) [10, 11]. These provide detailed and comprehensive classifications based on clinical diagnostic criteria [12, 13]. In this study, we are only concerned with sleep disturbances in general, in terms of insomnia, daytime sleepiness, and sleep quality.

Generally, daytime sleepiness is a difficulty in maintaining an adequate level of wakefulness [14]. Daytime sleepiness (DTS) is a common symptom of many sleep disorders (SSS). These disorders include periodic limb movement disorder (PLMD), obstructive sleep apnea syndrome (OSAS), idiopathic hypersomnia, narcolepsy, and other different disorders [15]. The definition of DTS is somewhat complicated. Some view sleepiness as a state of feeling tired or fatigued and describe it as the subjective feeling that comes before sleep onset as in the Stanford Sleepiness Scale (SSS) [16]. Others define it as a state of decreased alertness. Studies have demonstrated that 1 in 5 adults goes into episodes of sleep during wake time. Sleepiness that impairs daily functioning affects about 16% of adults [17].

Many methods have been established to measure daytime sleepiness such as the multiple sleep latency test (MSLT) [18, 19], the maintenance of wakefulness test (MWT) [20], and Epworth Sleepiness Scale (ESS) [21]. The latter is discussed in further details below.

Daytime sleepiness is highly prevalent among college students. Fifty-percent of university students describes daytime sleepiness and 70% obtain inadequate sleep [22].

Lack of sleep is highly correlated with low academic performance, decreased learning abilities, and reduced memory [23, 24]. Medeiros suggested that irregular sleep-wake cycle is linked to poor students performance in medical college [25, 26]. Rodriguez RN observed that students who did not get enough sleep did not perform well on their final examinations compared to the others [27].

Despite the large number of investigations that tackled the relationship between DTS and academic performance and the incidence of DTS among students in different parts of the world, this is the first study in Jordan that aimed to elucidate the prevalence of DTS in Jordan medical students and to determine the impact of sleep disorders such as DTS on academic performance. Thus, the aim of this study was to determine the prevalence of DTS among students from medicine and surgery, dentistry, nursing, pharmacy, and doctor of pharmacy colleges and to examine the impact of sleep disorders on students' academic achievement.

## 2. Methodology

Students enrolled to health colleges at Jordan University of Science and Technology (JUST), Irbid, Jordan, were targeted in this study. The period of the study was between November 1 and December 31, 2018. A cross-sectional questionnaire study was designed to answer the goals of the study easily and conveniently. The study was approved by JUST institutional review board (IRB). The health collages involved in the study were medicine and surgery, dentistry, applied medical sciences, nurses, pharmacy, and doctor of pharmacy (pharm D).

The questionnaire was distributed to the participants on the campus by the researcher. The voluntary participation in the study was emphasized, and all participant inquiries and concerns were addressed and clarified.

### 2.1. The Study Questionnaire

Experts in the field including public health specialist, philosopher, and a sleep specialist reviewed the study questionnaire to validate it, and it was adjusted based on pilot study feedback from 20 participants. Cronbach's *α* score for ESS was 0.73 which ensured the internal consistency of the study. The study questionnaire was created based on Epworth Sleepiness Scale (ESS) and similar designs in the literature [27]. The participants recorded their cumulative grade point average (CGPA) which was utilized to assess the students' academic performance. The aim of the questionnaire was to identify daytime sleepiness and demographic and other related variables in the students from the five targeted health collages. The students' responses were limited to events that happened during the last 30 days.

### 2.2. ESS

ESS is an index that measures daytime sleepiness based on the response of the subject to 8 questions that tackle sleep in real life. Each question is scaled on a 4-point scale from 0 to 3 yielding a total score from 0 to 24. Scores of 0-9 indicate normal ESS, 10-11 are border line, and 12-24 means that a medical consultation should be asked. Scores higher than or equal to 10 were considered abnormal [28]. It is a reliable and validated sleep questionnaire based on many reports [29, 30].

### 2.3. Data Analysis

Data entry, tabulation, and interpretations were done using the Statistical Package for Social Sciences (SPSS) software (version 22.0). *t* test and one-way analysis of variance (ANOVA) test were utilized to compare between and two groups and more than two categorial groups of ESS scores, respectively. Pearson chi-square was used to determine the statistical significance of any association between groups. Tukey post hoc tests were used to determine the differences between any pair of groups.

## 3. Results

### 3.1. Participants' Demographics

977 students with a mean age of 20.9 ± 2.2 participated in this study, and 616 of them were females and 361 were males. This distribution is consistent with the higher number of females in the university in general. Students were distributed on five schools with the majority of the students were from the school of Medicine, 299 (30.6%), followed by the school of allied medical sciences, 212 (21.7%). Participants from dentistry, nursing, and pharmacy were close in numbers, 159 (16.3%), 152 (15.6%), and 154 (15.8%), respectively.

The 6^th^ and 5^th^ year students represent 23.5% of all participants (the largest group of participants), while the 1^st^ year students were only 16.1% of the participants which represents the smallest group. Percentages of other years are as follows: 2^nd^ year is 22.5%, 3^rd^ year is 15.5%, and 22.5% from the 4^th^ year. A total of 22.4% of the students reported the use of sleep aid medication at least once/week in the previous month. Complete demographic profiling is presented in Table 1.

### 3.2. Daytime Sleepiness (ESS)

ESS questionnaire was used to examine the daytime sleepiness of the students. Having a score of 10 or above was used as the cut-off for abnormal ESS scores based on the literature review. The average ESS score (±SD) was 7.7 ± 4.6. ESS scores were normal in 629 students (65.6%), while 330 students (34.4%) were defined as abnormal and were considered to have daytime sleepiness as shown in Table 2. One item was related with a high chance of sleeping in 23% of the participant: “lying down to rest in the afternoon when circumstances permit”. Gender had no significant association with ESS scores (*p* = 0.12). However, academic year, specialization, insomnia, sleep quality, and hours of sleep had a significant association with ESS scores (*p* < 0.05) which are illustrated in Table 3 and interpreted as the following: academic year, second year students had a significant greater ESS mean scores compared to the sixth- and fourth-year students; specialization, student from the pharmacy scored significantly higher ESS mean scores than students from the medicine and surgery and had a significantly greater mean ± SD of ESS scores compared to the medicine and surgery students; sleeping hours, students who had less than 5.9 hours of sleep had significantly greater mean ± SD ESS scores than the students who had more than 7 hours; and sleep quality, good reported quality of sleep was associated with significantly lower ESS mean scores compared with the students with bad reported sleep.

### 3.3. Field of the Study and Other Sleep-Related Variables

Student's specialization was significantly associated with the presence of insomnia and number of night sleeping hours (Table 3). Pharmacy and pharm D students have the largest percentages of insomnia (81.8%). A total of 23.9% of dentistry students were sleeping less than 5 hours at night, while 36.2% of students from nursing and midwifery college were sleeping more than 7 hours at night (36.2%).

The use of sleeping pills was the most common among students from the pharmacy and pharm D college compared with the other students, and a total of 29.9% (*X*^2^ = 5.8, *p* = 0.02) of students from this collage reported the use of sleeping pills.

### 3.4. Cumulative Grade Point Average (CGPA)

There was no significant association between the disclosed CGPA and ESS scores (Table 4). However, CGPA was significantly associated with the reported sleep quality. A total of 63.3% students with a good sleep quality response had a CGPA of 3.5 and above (*p* = 0.02, Table 5). On the other hand, 55.6% of students with bad reported sleep quality had a CGPA of less than 2.5 (Table 5).

## 4. Discussion

This study demonstrates that only 26.1% of students get more than 7 hours of night sleep that a normal young individual should have [31]. On the other hand, 42% of the students indicated that they have less than 6 hours of night sleep. Our data was comparable to the results of a large cross sectional study conducted by the college students that showed that a quarter of students get more than 6.5 hours of nigh sleep and only about thirty percent of the students have equal or more than 8 hours of night sleep [32]. These results agree with the mean pooled sleep duration for medical students analyzed from 24 studies from 13 countries. The average actual sleep duration at night was 6.3 hours [33]. Interestingly, this meta-analysis study showed that 55% of all medical students included by these studies complaint of poor night sleep. In comparison with previous studies, students in this study have attained a higher percentage of reduced amount of sleep which might be attributed to several factors such as increased academic load. Perhaps future studies can address the reasons.

The result of the present study revealed ESS scores mean ± SD of 7.7 ± 4.6 which is less than what has been reported in other studies, such as in Brazil, which showed 10.0 ± 3.7 [34].

The current study illustrated that the DTS frequency among medical and paramedical students based on the ESS score was 34.4%. This could be the result of bad sleep at night, either insufficient duration or bad quality. The prevalence reported here is almost similar to those reported in Saudi Arabia (36.6%) [35] and Malaysia (35.5%) [36], and Ramamoorthy et al. reported 30.5% of medical students in India to suffer from daytime sleepiness [37] that was supported by another report from India with about 37% of the participants suffering from DTS [38]. On the other hand, many reports from Taiwan, USA, and Turkey daytime sleepiness was much lower than the prevalence reported here [39–41]. While the prevalence DTS among medical students in countries like Brazil and Latin America was higher [22].

Interestingly, DTS prevalence among medical students in Jordan in this reported is not very different from the prevalence of daytime sleepiness in the general population (5-30%) [33].

These differences could reflect cultural and socioeconomic differences between medical student from different countries that leads to changes in their sleep patterns. However, this speculation needs scientific proof.

No gender dominance was found between ESS score and gender in our study which was different to what was reported by other studies. For instance, in Abdulghani study, it was shown that females tend to have a higher ESS score in comparison to males [35, 36].

The current results illustrated that self-reported bad quality of sleep was associated with abnormal ESS scores which are analogous to what was reported by Zailinawati et al. [36]. Alseggaf et al. reported that EES scores were a good estimate of self-reported quality of sleep among medical students in clerkship years [42].

The impact of sleeping disorders on academic performance has been addressed by many studies. Rasekhi et al. reported that abnormal PSQI scores were associated with lower academic achievement [43]. Abdulghani et Al. and Arbinaga et al. reported that there is a strong relationship between the good academic performance and the quality of sleep [35, 44, 45]. This report supports these observations. It clearly showed that good academic performance among medical student in Jordan as inferred from CGPA was dependant on good sleep quality. However, in a very closely related community in Palestine, there was no association between CGPA and the quality of sleep which could indicate the special conditions of the Palestinian community [46].

This study reported no significant association between CGPA and ESS scores which is incongruent with what was found with Abdulghani et al. who reported a statistically significant association between academic achievement and ESS values.

## 5. Limitations

Finding from this study should be interpreted with the following limitations in mind. First, CGPA was self-reported by the participants which decreases the objectivity of the conclusions drawn from the study. Second, data were from one university in Jordan; thus, results may not be generalized to the whole country. Last, sleep problems could arise from variety of causes other than the academic loads. This study did not address the causes of daytime sleepiness. Understanding and investigating these causes among medical students may change the interpretations of sleep quality and academic performance and thus limit the current study conclusions.

## 6. Conclusion

Daytime sleepiness is highly prevalent among medical, dental, nursing, paramedical, and pharmacy students in Jordan. Although the prevalence of DTS among Jordanian medical students is lower than some countries like Latin America and Brazil and higher than what is reported in other countries like USA, Taiwan, and India, it falls within the average prevalence of the general population. As the academic performance depends on the sleep quality, it is very important to educate the college students regarding the practice of good sleep hygiene and quality.

Additionally, we encourage the educational policy makers in medical education to study the causes of high sleep problem in Jordan medical students and to investigate the impact of this important disturbed behavior on other student-related variable such as mental health.

## Figures and Tables

**Table 1:** Participant's demographic profile (n = 986).

No.	Variable	Statistics ∗
1	Age	Mean 20.9 ± 2.2
2	Sex	
	Female	616 (63.1%)
	Male	361 (36.9%)
3	School	
	Medicine	299 (30.6%)
	Allied medical sciences	212 (21.7%)
	Pharmacy and pharm D	154 (15.8%)
	Dentistry	159 (16.3%)
	Nursing and midwifery	152 (15.6%)
4	Year of study	
	1st	156 (16.1%)
	2nd	218 (22.5%)
	3rd	150 (15.5%)
	4th	218 (22.5%)
	5th	120 (12.4%)
	6th	108 (11.1%)
5	Medical problem	
	Yes	109 (11.2%)
	No	868 (88.8%)
6	Night sleeping hours	
	<5	155 (15.9%)
	5-5.9	256 (26.3%)
	6-6.9	310 (31.8%)
	>7	254 (26.1%)
7	The use of sleep aids	
	0 in the past month	757 (77.6%)
	< once/week	103 (10.6%)
	Once or twice/week	73 (7.5%)
	>3 times/week	43 (4.4%)
8	Quality of sleep	
	Good	570 (58.8%)
	Bad	399 (41.2%)
9	CGPA	
	Below 2.5	45 (4.9%)
	2.5 - 2.99	202 (26.7%)
	3 - 3.49	321 (34.7%)
	3.5 and above	358 (38.7%)

∗Percentages represents the frequency for categorial variables and the means ± SD for continuous variables. 6^th^ year students are from the colleges of Doctor of Pharmacy (Pharm D) and Medicine and Surgery.

**Table 2:** ESS scores values and their interpretations.

No.	Variable	Statistics ∗
1	ESS scores	7.7 ± 4.6
	Normal (0-9)	629 (65.6%)
	Abnormal (≥10)	330 (34.4%)
2	ESS scores interpretation	
	Normal (0-9)	629 (65.6%)
	Borderline daytime sleepiness (10-11)	118 (12.3%)
	Medical advice should be sought (12-24)	212 (22.1%)

∗ Percentages represents the frequency for categorial variables and the means ± SD for continuous variables.. ESS: Epworth Sleepiness Scale.

**Table 3:** Association between field of study and sleep-related variables. Table 3 chi-square results of the association between the specialty of the student and tested other categorial. Bolded numbers represent the highest percentages.

	School of Nursing	School of Pharmacy	School of Dentistry	School of Applied medical sciences	School of Medicine and surgery	Chi-square	p value
ESS scores							
Normal	62.4%	60.3%	**70.3%**	63.6%	68.7%		
Abnormal	37.6%	**39.7%**	29.7%	36.4%	31.3%	5.7	0.22
Total	100%	100%	100%	100%	100%		
Night sleeping hours							
<5	14.5%	19.5%	**23.9%**	12.8%	12.8%		
5-5.9	21.1%	27.9%	25.8%	26.5%	28.2%	38.6	< 0.001
6-6.9	28.2%	34.4%	31.4%	26.1%	36.5%		
>7	**36.2%**	18.2%	18.9%	34.6%	22.5%		
Total	100%	100%	100%	100%	100%		
Sleep quality							
Good	**62.5%**	59.1%	55.8%	61.9%	56.1%	3.2	0.53
Bad	37.5%	40.9%	**44.2%**	38.1%	43.9%		
Total	100%	100%	100%	100%	100%		
Using sleeping pills							
No	80.3%	70.1%	**83.0%**	75.4%	78.6%		
Yes	19.7%	**29.9%**	17.0%	24.6%	21.4%	9.01	0.06
Total	100%	100%	100%	100%	100%		

**Table 4:** Total ESS scores with students' characteristics. Table 4: represents the statistical analysis of the total ESS scores with students' characteristics analyzed by the independent samples t-test and ANOVA test.

	Total ESS value score		
		The median	
	(mean ± SD)	Min-max	p
Sex			
Female	7.5 **±** 4.5	7.0 (0-24)	0.12
Male	8.0 **±** 4.5	8.0 (0-24)	
Academic year			
_1_st	7.7 **±** 4.4	7.0 (0-22)	<0.05
_2_nd	8.5 ± 4.4	9.0 (0-23)	
_3_rd	7.8 **±** 4.4	7.0 (0-18)	
_4_th	7.1 **±** 4.3	7.0 (0-24)	
_5_th	7.7 **±** 4.5	8.0 (0-18)	
_6_th	7.1 **±** 5.3	7.0 (0-24)	
College			
Medicine	7.2 ± 4.7	7.0 (0-24)	<0.05
Pharmacy & PharmD	8.6 ± 4.8	8.0 (0-23)	
Dentistry	7.9 ± 4.1	8.0 (0-22)	
Applied medical science	7.8 ± 4.4	8.0 (0-17)	
Nursing	7.6 ± 4.5	7.0 (0-22)	
Presence of insomnia			
Insomnia	8.3 ± 4.6	8.0 (0-24)	<0.05
No insomnia	5.8 ± 3.8	5.0 (0-18)	
Sleep quality			
Good	7.0 ± 4.2	6.0 (0-22)	<0.001
Bad	8.8 ± 4.8	9.0 (0-24)	
Sleeping hours			
>7	7.0 ± 4.3	7.0 (0-23)	<0.05
6-6.9	7.3 ± 4.2	7.0 (0-22)	
5-5.9	8.6 ± 4.7	9.0 (0-24)	
<5	8.5 ± 4.9	8.0 (0-24)	
CGPA			
>3.5	7.43 ± 4.6	7.0 (0-24)	**0.495**
3-3.49	7.97 ± 4.5	8.0 (0-24)	
2.5-2.99	7.82 ± 4.4	8.0 (0-24)	
<2.5	8.11 ± 5.7	8.0 (0-22)	

**Table 5:** Self-reported sleep quality and CGPA, number of students responses.

CGPA	GOOD	Bad
<2.5	19	26
2.5-2.99	123	78
3-3.49	175	146
>3.5	226	131

## Data Availability

The datasets used and/or analyzed during the current study are available from the corresponding author on reasonable request.
